# Non-Coding RNAs and Splicing Activity in Testicular Germ Cell Tumors

**DOI:** 10.3390/life11080736

**Published:** 2021-07-24

**Authors:** Marco Barchi, Pamela Bielli, Susanna Dolci, Pellegrino Rossi, Paola Grimaldi

**Affiliations:** Department of Biomedicine and Prevention, University of Rome “Tor Vergata”, 00133 Rome, Italy; marco.barchi@uniroma2.it (M.B.); pamela.bielli@uniroma2.it (P.B.); dolci@uniroma2.it (S.D.); pellegrino.rossi@med.uniroma2.it (P.R.)

**Keywords:** sncRNA, lncRNA, splicing, rRNA, R-loop, testicular germ cell tumor

## Abstract

Testicular germ cell tumors (TGCTs) are the most common tumors in adolescent and young men. Recently, genome-wide studies have made it possible to progress in understanding the molecular mechanisms underlying the development of tumors. It is becoming increasingly clear that aberrant regulation of RNA metabolism can drive tumorigenesis and influence chemotherapeutic response. Notably, the expression of non-coding RNAs as well as specific splice variants is deeply deregulated in human cancers. Since these cancer-related RNA species are considered promising diagnostic, prognostic and therapeutic targets, understanding their function in cancer development is becoming a major challenge. Here, we summarize how the different expression of RNA species repertoire, including non-coding RNAs and protein-coding splicing variants, impacts on TGCTs’ onset and progression and sustains therapeutic resistance. Finally, the role of transcription-associated R-loop misregulation in the maintenance of genomic stability in TGCTs is also discussed.

## 1. Introduction

Testicular germ cell tumors (TGCTs) are the most frequent solid tumors of adolescents and young adult men (between 15 and 35 years of age). They are a heterogeneous group of neoplasms that includes three types of tumors occurring at distinct ages: pre-pubertal TGCTs, comprising teratomas–yolk sac tumors in boys younger than 12 years of age (Type I), post-pubertal TGCTs, comprising seminomas and non-seminomas in young men between 15 and 40 years of age (Type II) and spermatocytic seminomas that generally are present in men older than 50 years of age (Type III) [1,2,3].

By far, the most prevalent subtype is Type II post-pubertal TGCT, with a lifetime risk of about 0.5–1% that in some countries has increased up to three-fold in the last five decades [2,4]. Type II TGCTs can be divided clinically and histologically into two main types: seminomas (SGCT), that are mainly homogeneous germ cell tumors composed of embryonic germ cells, and non-seminomas (NSGCT), the most aggressive heterogeneous group of tumors which arises at various stages of embryonic differentiation ranging from embryonal carcinoma (ECs), choriocarcinoma (CH) and yolk sac tumor (YST) to the more mature teratoma type [5]. 

All TGCTs are thought to originate from a common precursor, the germ cell neoplasia in situ (GCNIS) [6]. GCNIS cells stay quiescent during infancy followed by proliferation in puberty, probably due to hormonal stimulation, with subsequent progression into tumors. The biological causes of the initial malignant transformation from a precursor to GCNIS cells are still unclear. The initial transformation most likely takes place in utero during the early development of the germline and the target cells are most likely the embryonic germ cells, either primordial germ cells (PGCs) or gonocytes [7,8,9,10]. It is likely that multiple factors contribute to the development of TGCTs and that both genetic and environmental factors play a pivotal role in tumorigenesis. Beyond age, race, and family history of the disorder, strong risk factors are cryptorchidism [11], infertility [12] or genetic conditions such as Down’s syndrome [13], Klinefelter’s syndrome [14] or XY gonadal dysgenesis [15], suggesting that inherited factors or congenital genetic changes are associated to TCGTs.

Although several studies have tried to identify the molecular mechanisms underlying TGCT development, they are still poorly known. Multiple lines of evidence reported that altered RNA metabolism is one potential cause involved in the pathogenesis of several human tumors including TGCTs [16]. RNA metabolism includes regulation, modification and stability of transcripts and involves a broad class of RNA molecules with different functions, classified as coding and non-coding RNAs. Coding molecules are represented by messenger RNAs (mRNA), while non-coding molecules (ncRNA) include different other RNA species with regulatory functions. The production of functional mature messenger RNA (mRNA) is dependent on different regulatory processes involving non-coding RNAs and RNA binding proteins. In this context, RNA metabolism defects induce detrimental effects on physiological cellular processes, promoting pathological conditions. In this review, we present the state of art of aberrant RNA metabolism in the development of TGCTs.

## 2. Non-Coding RNA

**Figure 1:**
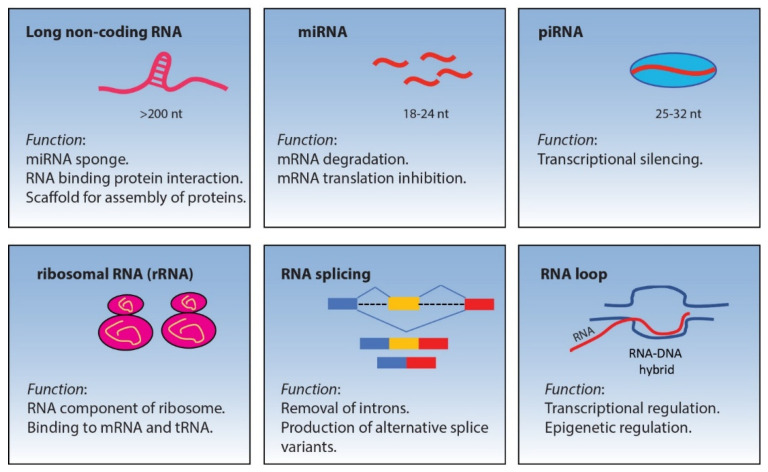
Schematic representation of non-coding RNAs and their biological functions.

Noncoding DNA covers 95% of DNA sequences in the human genome, most of which are transcribed into various non-coding RNAs (ncRNAs) species which play a role in regulating gene expression. Non-coding RNAs can be classified based on their size. Those having a size less than 200 bp are called small non-coding RNAs (sncRNAs) while those above 200 bp are called long non-coding RNAs (lncRNAs) [17]. Examples of sncRNAs include microRNA (miRNAs), small interfering RNAs (siRNAs), Piwi-interacting RNAs (piRNAs) and small nucleolar RNAs (snoRNAs). Figure 1 reports a list of ncRNAs involved in TGCTs and their biological functions. 

Testes express large numbers of ncRNAs, mainly miRNAs, piRNAs and lncRNAs that play a role in the regulation of gene expression. They are expressed in a cell-specific manner during spermatogenesis, regulating gene expression at each step of male germ cell differentiation [18,19]. A large body of evidence indicates that deregulation of ncRNAs’ expression is potentially involved in testicular germ cell tumorigenesis. This hypothesis is also supported by the observation that many of the risk loci in TGCT are in the non-coding regions of the genome [20].

### 2.1. miRNA

MicroRNAs (miRNAs) are the most abundant class of sncRNAs, ranging between 18–24 nucleotides. They are generated from transcribed hairpin loop structures and regulate gene expression at transcriptional and post-transcriptional levels. They are involved in several biological processes, including cycle, proliferation, differentiation and apoptosis. MiRNAs are transcribed by RNA polymerase II and exert their function by targeting ~60% of the transcripts from the human genome. They bind to 3′-untranslated regions (UTRs) of mRNA targets in a sequence-specific manner, negatively regulating transcript stability and translation. A single miRNA can potentially interact with many mRNAs and a single mRNA can be bound by various miRNAs [21].

MiRNAs are frequently dysregulated in several human cancers and can act as oncogenes or tumor suppressor genes [22]. Several observations have highlighted the importance of miRNAs also in TGCT development. Aberrant miRNA expression profile has been well documented in TGCTs compared to normal testis [23,24] and, interestingly, miRNAs seem to vary in different histological subtypes of TGCTs. However, very few miRNAs have been functionally characterized in these tumors [25].

Genes encoding for miRNAs are located throughout the genome, and a large proportion are found organized within clusters comprising multiple miRNAs. Two clusters, miR-302/367 and miR-371-373, seem to be dysregulated consistently in TGCTs and their high expression indicates that they may act as oncogenes.

The miR-302/367 cluster consists of five pre-miRNAs: mir-302b, mir-302c, mir-302a, mir-302d (from the mir-302 family), and mir-367. This cluster plays a role in the maintenance of embryonic stem cells pluripotency and its overexpression promotes cellular reprogramming and maintains the stemness of human embryonic stem cells (hESCs) [26,27]. At the molecular level it has been demonstrated that miRNA-302/367 promotes proliferation and accelerates G1 to S transition of the cell cycle by targeting the Rb family and CDK1NA [28]. In EC cell lines NT2-D1 and 833 K, a high level of miR-302s positively associates with expression of SPRY4, a regulator of MAPK/ERK and PI3K/Akt signaling pathways. Interestingly, EC treatment with cisplatin induced a downregulation miR-302s and, concomitantly, a reduction in SPRY4 expression level. Furthermore, inhibition of miR-302b-3p and miR-302c-3p decreased phosphorylation of ERK1/2, suggesting that miR-302s may act as oncogene inducing the expression of SPRY4 and activating MAPK/ERK pathway in EC cell lines [29].

The miR-371-3 locus represents one of the main miRNA clusters involved in TGCT tumorigenesis. In a functional genetic screen, miR-372 and miR-373 have been shown to be upregulated in TGCT and to act as oncogenes promoting cell proliferation and tumor development. The underlying molecular mechanism relies on repression of tumor suppressor LArge Tumor Suppressor homolog 2 (LATS2). Indeed, binding of miR-372 and miR-373 to 3′UTR suppresses protein translation of LATS2 mRNA. LATS2 is a serine-threonine kinase whose deletion accelerates cellular proliferation and tumor development in flies [30] and mice [31]. In line with miR-372 and miR-373 activity, it has been reported that downregulation of LATS2 protein correlates with sustained activity of CDKs and uncontrolled cell proliferation [25].

Using published dataset miR-223-3p has been found upregulated in TGCT compared with normal testes. MiR-223-3p exerts oncogenic role in TGCT through repression of FBXW7, a substrate-recognition component of the SCF-ubiquitin-ligase complex, a tumor suppressor that targets multiple oncoproteins and oncogenic transcription factors for ubiquitination-mediated proteolysis [32]. Furthermore, miR-223-3p upregulation promotes cell proliferation and inhibits apoptosis. Notably, Liu et al. [33] reported an inverse correlation between miR-223-3p and FBXW7 mRNA expression in TGCT. High levels of miR-449a-b have been also found in normal testes, lung, and trachea while they are strongly downregulated in several cancer cells including testicular cancer. According with a putative tumor-suppressive role, miR-449a strongly promotes apoptosis and upregulates p53 activity, reducing cell proliferation. MiR-449a and miR-449b are highly upregulated by the transcription factor E2F1 that is essential for cell proliferation. MiR-449a can reduce the expression of cell cycle protein CDK6, thereby counteracting cell cycle progression. [34].

Downregulation of another cluster, miR-506~514, has been reported in seminomas and EC. In in vitro functional studies, Özata et al. [35] provided evidence that loss of miR-514a-3p in TGCT increases the expression of its target gene Paternally Expressed Gene 3 (PEG3). Since PEG3 activates the NF-κB pathway, protecting cells from apoptosis, it has been suggested that this molecular mechanism could be involved in TGCT development.

De Martino et al. [36] have demonstrated a potential role of Let-7a and miR-26a miRNAs in seminomas. By using the Cancer Genome Atlas database, Let-7a and miR-26a have been found to be downregulated and negatively correlated with *HMGA1* expression levels in human seminoma. In functional studies using human seminoma cell line TCam-2, the authors also reported that Let-7a and miR-26a play a critical role in inhibiting seminoma cell growth and motility by directly regulating HMGA1 expression [36]. Recently, it has been suggested that miRNA dysfunction in tumor cells can also modulate the tumor microenvironment, such as angiogenesis, immune cell recruitment and metastasis [37,38,39]. It has been reported that miR-125b is downregulated in TGCT and that low levels are associated to increased production of tumor-derived chemokine CSF1 and CX3CL1, which are known to control the recruitment of macrophages to the neoplastic sites that in turn stimulate tumor growth [40].

Other miRNAs such as miR-99a, miR-100 and miR-145 are reported to be downregulated or upregulated, such as miR-512-3p, miR-515, miR-517~518 and miR-525, in TGCTs, but their functional role has not been still elucidated [41].

Recently, miRNAs have gained an important role in the diagnosis and treatment of cancer. Indeed, miRNAs are released into biological fluids by testicular cancer cells and can be detected in blood and serum from affected patients [42]. Their abundance in serum and the relative easiness of detection makes them suitable as potential tumor markers.

### 2.2. piRNA

piRNAs are single-stranded RNAs which range from 23 to 32 nt. In mammals, these RNAs are expressed mostly in male germ cells, although also bovine oocytes express functional piRNAs [43,44]. Initially identified as repeat-associated siRNAs (rasiRNAs), piRNAs also match transposable elements and associate to Argonaute proteins that named them as pi-RNAs after PIWI, the founder of the PIWI-clade [45,46,47]. Genomic loci encoding for piRNAs are organized in clusters and harbour transposon fragments, providing a genetic record of past transposition events. PiRNA-associated proteins are expressed in male and female germ cells and their function is essential for maintaining the genomic integrity of the germline [43,44,48]. Hypermethylation of PIWI pathway genes and a concomitant decrease of their expression levels have been demonstrated in seminomas compared to healthy testes, suggesting a role of PIWI and piRNAs in TGCT tumorigenesis [49,50]. In agreement, deep sequencing data of small RNAs in GCNIS and TGCTs samples revealed that the loss of piRNAs is a hallmark of TGCT samples [51]. In the search of early biomarkers of testicular cancer, several classes of small non-coding RNAs have been reported to be present in seminal plasma from healthy donor males, including piRNAs. Attempts have been made to link the presence of specific piRNA classes to TGCTs in order to predict the occurrence of either TGCT or GCNIS. However, larger cohorts of samples are needed to validate the diagnostic potential of these RNAs in seminal plasma [52].

### 2.3. lncRNA

LncRNAs are conventionally defined as transcripts with lengths exceeding 200 nucleotides that are not translated into protein. LncRNAs function lies in their capacity to bind and regulate a molecular partner either via base-pair interactions or through their secondary structure. After binding their partners, lncRNAs can act as baits (molecular sponges), guides and scaffolds for the assembly of ribonucleoprotein complexes or can act as allosteric regulators [53,54]. LncRNAs have been implicated in diverse biological processes, and often they are found to be highly deregulated in tumors, in which they can act as either tumor suppressors or oncogenes [55]. Recently the possible role of lncRNAs in TGCT has started to emerge [56].

The first link between lncRNA and TGCTs was found for the transcript of the XIST gene, which was found to be expressed in TGCTs following the acquisition of supernumerical X chromosomes [57]. More recently, XIST expression and the XIST-promoter demethylation status have been proposed as tissue biomarkers for TGCTs, which could discriminate between seminomas and non-seminomatous tumors [58].

One of the best-characterized lncRNA associated to TGCTs is the transcript of the Testis Developmental-Related Gene 1 (TDRG1). TDRG1 was found to be upregulated in seminoma, in which promotes tumor growth, progression and chemoresistance to cisplatin [59,60,61,62].

The expression of lncRNA TDRG1 in TGCTs has been shown to be linked to the expression of another lncRNA, H19. This lncRNA is encoded by a paternally imprinted gene, which is normally expressed only by the maternal allele in males. H19 was found to be upregulated in TGTCs, probably due to loss of imprinting, which possibly reflects TGCT formation during early stages of embryogenesis, when biallelic expression of H19 normally occurs [63,64,65,66,67].

It has been proposed that that H19 promotes the expression of TDRG1 in the established cisplatin-resistant TCam-2 cell line by acting as a molecular sponge sequestering miRNA-106b-5p, which normally negatively regulates TDRG1, thus facilitating cell survival in cisplatin-based chemotherapeutic conditions [68].

SPRY4-IT1 is an lncRNA acting as a miRNA sponge that positively regulates several oncogenic signalling pathways in melanoma [69]. A recent paper showed that high expression levels of SPRY4-IT1 are detected in human TGCTs, and that transient knockdown of SPRY4-IT1 in two TGCT cell lines resulted in decreased cell growth, migration and invasion, concomitant to a significant reduction in the phosphorylation of Akt [70].

NLC1-C, also known as long intergenic non-protein-coding RNA162 (LINC00162), was found to be downregulated in the cytoplasm and accumulated in the nucleus of spermatogonia and primary spermatocytes in the testes of infertile men with mixed patterns of maturation arrest compared with normal controls. In the same paper, the authors show that accumulation of NLC1-C in the nucleus promoted proliferation of a testicular embryonal carcinoma cell line by binding to Nucleolin and thus repressing transcription of miR-320a and miR-383, two miRNAs with known oncosuppressive roles that, in turn, negatively regulate NLC1-C expression [71].

HOXA transcript at the distal tip (HOTTIP) is a 3764 nucleotide long non-coding RNA (lncRNA) encoded from a genomic region within 5′ of the HOXA locus, and it is considered as an oncogenic lncRNA and a potential biomarker and therapeutic target in almost all kinds of human cancers [72]. HOTTIP was found to be highly expressed in the testicular embryonal carcinoma cell line NT2, in which its knockdown impaired cell proliferation, whereas its overexpression promoted it. In the same paper, HOTTIP was proposed to act as a sponge for miR-128-3p, a miRNA with anti-proliferative action, thus positively modulating expression of the oncogenic transcription factor HOXA13 [73].

### 2.4. Ribosomal RNA (rRNA)

Ribosomes are very large ribonucleoprotein particles (RNPs) that in humans consist of at least 4 ncRNAs (18 S, 28S, 5S and 5.8S) and more than 80 associated proteins defined as ribosomal proteins (RPs), organized in two subunits. The small ribosomal subunit (40S) contains the 18S rRNA and 33 RPs, and the large subunit (60S) contains the 5S, 5.8S and 28S rRNAs and about 47 RPs.

Further complexity to the ribosomal system is added by the discovery that human cytosolic rRNA contains 14 distinct types of post-transcriptional modifications in 228 sites [74] that control the translational fidelity or the choice of translation initiation (i.e., CAP versus internal ribosome entry site (IRES)) of key tumor suppressor genes [75]. rRNA is the catalytic core of the complex and RPs are the structural units that help to organize its ribozyme activity [76]. DNA sequences encoding for rRNAs (rDNA) are distributed along the short arms of all human acrocentric chromosomes and form extended loops that define the nucleolar organizer regions (NORs) visible at the microscopic level as nucleoli. rDNAs are transcribed by RNA polymerase I (Pol I) to generate pre-rRNAs that, through several processing steps, produce mature 18S, 5.8S and 28S rRNAs. RNA Polymerase III directs 5.8S rRNA synthesis. The rDNA locus is the most highly transcribed region of the eukaryotic genome, and Pol I activity accounts for more than 60% of total cellular transcription, making Pol I activity essential for cell growth and proliferation [77].

Defects in rRNA production result in different human ribosomopathies and cancer transformation. In cancer cells, uncontrolled rRNA synthesis can occur by overactivity of oncogenes such as Myc or loss of tumor suppressors such as p53, Arf or pRb. [78,79,80,81] and such metabolic activity is linked to the increase of number and size of nucleoli, a typical feature of cancer cells. In this context, it has been observed that TGCTs as well as GCNIS are characterized by enlarged hyperchromatic nuclei, clumped chromatin and, often, prominent nucleoli. Ectopic NORs have been described in primary germ cell tumors and in teratocarcinoma cell lines that could have been originated by chromosomal rearrangement or by derepression of preexisting inactive NORs [82,83]. All together these features indicate that rRNA synthesis is a potential target process for anti-cancer therapy and that inhibition of Pol I represents an attractive therapeutic approach to block ribosome biogenesis [84]. Indeed, Pol I inhibition induces ribosomal stress and through p53 dependent and independent pathways causes cell death, cell cycle arrest or senescence. Recently, two promising specific inhibitors of Pol I activity (CX-5461 and BHM-21) have been developed [85,86] and one of them (CX-5461) has passed phase I clinical trial (NCT02719977 and NCT04890613). To this end, we identified a natural molecule, sempervirine, extracted from *Gelsemium sempervirens* and previously thought to act as an MDM2 inhibitor [87] that specifically targets Pol I in TGCTs [88]. We found that this molecule binds and disassembles nucleoli of tumor cells after few hours of culture and induces PolI degradation, leading TGCT cells, but not non-tumor cells, to p53 dependent and independent cell death.

## 3. RNA Splicing

The discontinuity of eukaryotic genome requires that introns are spliced out and exons joined to form a continuous RNA transcript. However, certain exons deviate from this regulation resulting alternatively included in the mature mRNA. This process, known as alternative splicing (AS), allows yielding multiple transcripts from the same gene expanding the proteome diversity and complexity of the genome. This process is catalysed by the spliceosome, a ribonucleoprotein complex, assisted by auxiliary splicing factors [89]. Genome-wide analyses have revealed how this process is highly coordinated during cell differentiation and tissue development [90], proving to be a powerful means to finely tune gene expression during fundamental biological processes. Among organs, AS is particularly widespread in testis relying on activation of specific RNA processing programs that contributes to temporally regulate expression of genes essential for proper development of male gametes [91,92,93,94,95]. This suggests that splicing abnormalities might contribute to the development of pathological conditions. Accordingly, aberrant expression of splicing factors, as SF1, QK1 and RBFOX RNA-binding proteins, influences TGCTs’ incidence [96,97,98]. Furthermore, genome-wide profiling of TGCT datasets retrieved from The Cancer Genome Atlas revealed that numerous AS events are significantly associated with risk of disease progression [99]. Collectively, these observations point out AS dysregulation as a potential key driver that could promotes acquisition of hallmark traits in TGCTs, underpinning specific oncogenic processes and/or contributing to adaptive resistance toward chemotherapeutic agents.

In TGCTs, a large number of apoptosis- and cell cycle-related factors have been found to be regulated via AS [100,101]. The Ser/Thr-kinase NEK2 is a new key player regulating the interplay between splicing and cellular signalling [100,102]. In TGCTs, overexpression of NEK2 has been documented [103,104] and previous observations reported its activation during G2/M progression of male germ cells and its involvement in chromatin condensation during the meiotic divisions of mouse spermatocytes [105,106]. Recently, identification of NEK2 splicing isoforms allowed the identification of a novel function for this kinase, adding another layer of complexity with regard to its oncogenic activity [102]. NEK2 AS results in the differential expression of three isoforms. The canonical splice variant NEK2A results localized in the centrosome, and partially also in nucleus and cytoplasm, whereas NEK2B and NEK2C variants showed cytoplasmic and nuclear localization, respectively. In TGCTs, NEK2 was found enriched in the nucleus of several cancer cells, including testicular seminomas, where it interacts with and phosphorylates numerous splicing factors, including the oncogenic SR protein SRSF1. Notably, NEK2-mediated phosphorylation increases SRSF1 splicing activity toward its apoptotic target genes, promoting antiapoptotic variants of BCL-X, MKNK2 and BIN1. Accordingly, knockdown of NEK2 favoured pro-apoptotic splice variants, promoting apoptosis and sensitizing cells to chemotherapeutic treatment with cisplatin [102]. Besides NEK2, immunohistochemistry studies on seminomas and normal testis tissues also reported the cellular mis-localizzation of MAD2, a mitotic factor that act as a component of spindle assembly checkpoint (SAC) [107]. AS of MAD2 transcripts yields three isoforms, full length MAD2α, and MAD2β and MAD2γ, lacking exon 3 and exon 2 and 3, respectively. MAD2α was present in both the nucleus and the cytoplasm, while MAD2γ mainly localized to the nucleus and reduced mitotic arrest. Interestingly, in TGCTs patients, the overexpression of endogenous MAD2γ, but not MAD2α, was associated with resistance to cisplatin-based chemotherapy [108,109]. Since Nek2 interacts with MAD2 [110] and SAC has emerged as a promising target for cancer therapy [111], modulation of NEK2 activity and/or MAD2 AS could be exploited therapeutically in TGCTs patients displaying resistance to cisplatin-based chemotherapy. Accordingly, it has been shown that depletion of MAD2 induces apoptosis and restores sensitivity to cisplatin therapy in a cisplatin resistant lung cancer model [112]. Another gene that undergoes AS modulation, representing a valuable therapeutic target in cisplatin-resistant TGCTs, is the member of the inhibitor of apoptosis protein (IAP) family, Livin [100]. Although its expression in normal testicular tissue is still elusive [100,113,114], in a large cohort of TGCT patients the expression of both Livin α and Livin β splice variants was found to be strongly related to the histological subtype, resulting in frequently expressed seminoma [100]. Since Livin has been identified as a target for immune-mediated tumor destruction [115], clarifying the involvement of these isoforms in drug resistance might provide a therapeutic option for cisplatin-resistant patients.

In addition to evasion of apoptosis, metabolic adaptation, also known as aerobic glycolysis (or the Warburg effect), is an emerging hallmark of cancer [116]. Glycolytic key regulators have been found differentially expressed in TGCTs and associated with tumor aggressiveness [117,118], highlighting the potential role of metabolic adaptation among plausible causative processes of tumor progression. The contribution of AS to metabolism reprogramming has been reported in several tumors [119]. Notably, screening performed to find novel cancer-associated immunogenic gene products allowed the identification of four cancer-restricted splice variants of testis Lactate Dehydrogenase C (LDHC) in several cancers [117]. Three splice variants skip exons encoding NAD binding domains and/or L-lactate dehydrogenase active sites, thus yielding aberrant LDHC proteins devoid of specific enzymatic activity, whereas one isoform encodes for a fully active enzyme [117]. Lactate dehydrogenase catalyses the interconversion of lactate and pyruvate in the glycolytic pathway, and lactate has a pivotal role in spermatogenesis [120] and exerts an antiapoptotic effect [121]. Interestingly, a gene expression profile identified LDHC among genes differentially expressed in seminoma samples [122]. Although the impact of LDHC variants has not been investigated in TGCTs, these protein isoforms might exhibit different functional properties in terms of substrate specificity that may be beneficial for the metabolic adaptation, survival and proliferation of tumor germ cells.

### R-Loop

R-loops are three-stranded nucleic acid structures characterised by a DNA:RNA hybrid and a displaced single-stranded DNA that frequently form in connection with transcription. Their programmed formation occurs physiologically, and it contributes to important cellular processes including transcription initiation and termination, immunoglobulin class switching, replication of mitochondrial DNA and epigenetic modifications [123,124,125]. Besides their role in normally replicating cells, a large body of evidence suggests that mis-regulated formation of R-loops occurs in cancer cells. Unscheduled formation of R-loops is associated with transcription elongation defects, hyper-recombination and DNA damage, all of which might contribute significantly to cancer-related genome instability [125]. New technology, including the application of the S9.6 antibody, has led to the identification of R-loops interactome and knowledge of genome-wide distribution of R-loops [126,127,128]. One such RNA:DNA hybrid binding protein is Senataxin [128], a putative RNA:DNA helicase whose mutation is responsible for rare neurological disorders [129]. As deduced from GEO expression data and experimental results in mouse [130], Senataxin is highly expressed in testis, and its mutation in humans [131] or deletion in mice [132] causes germ cell arrest at pachynema, and unscheduled formation of R-loops in spermatocytes, likely as consequence of a conflict between transcription and meiotic recombination intermediates [132]. R-loops also accumulate in proliferating cells of Setx-/- mice testes [130,132], indicating a role for the helicase in resolving R-loops that occur in the germ cell lineage, due to the collision of the replication fork and the transcriptional machinery, as previously described in mitotic somatic cells [132,133,134]. Notably, double strand breaks (DSBs) induced by topoisomerase I treatment augment R-loops accumulate in proliferating germ cells of the testis, in both wild type and Setx-/- mice [130]. Since R-loops normally occur at pause sites during transcription [135], this indicates that DNA lesions can stall the transcription machinery, which in turn causes R-loop accumulation. This is further confirmed by the accumulation of R-loops in mitotic germ cells of the testis in mice deleted for Atm or Tdp1, two DNA-damage response genes required for repair of DNA breaks [136,137,138]. In this regard, it has been also demonstrated that defects in the homologous recombination (HR) proteins BRCA1 and BRCA2 [139,140], in the nucleotide excision repair (NER) proteins XPG and XPF [141] and in the Fanconi anemia (FA) pathway [142,143,144], lead to R-loop accumulation, thus indicating that several DNA repair pathways contribute to R-loop regulation [145].

To date, there are no reports in literature investigating R-loop levels in testicular germ cell tumors. Thus, whether an alteration of formation, stabilization or resolution of R-loops associates with TGCTs development is unknown. According to the exquisite sensitivity of TGCTs to cisplatin-induced damage, we demonstrated that embryonal carcinoma TGCT cell lines that are sensitive to drug treatment have a reduced proficiency of DSBs repair by HR [146]. In addition, analysis of NER protein expression in TGCT-derived cell lines revealed that levels of XPA, ERCC1 and XPF DNA repair proteins are reduced with respect to somatic tumor cells [146,147], indicating that repair of DNA damage by NER might be compromised. Moreover, in a recent study from our laboratory, in which EC cell lines sensitive and resistant to cisplatin were compared, we demonstrated that cisplatin-sensitive cell lines have a reduced expression of FANCD2 with respect to cisplatin-resistant cell lines [148]. Collectively these observations suggest that often, TGCTs have a reduced recombinative efficacy, which is one of the mechanisms that have been proposed to account for their unique sensitivity to DNA damage [149]. Given the importance of HR, NER and FANC pathways’ deficiency in R-loop accumulation in mitotic cells, it is plausible that R-loops might arise in replicating germ-cell tumor cells, contributing to increase genome instability and tumor progression. In accordance with this hypothesis, studies on TGCT tissues aimed at evaluating the activation of the DNA-damage response (DDR) in TGCTs have shown that DDR is not activated in pre-invasive carcinoma in situ (CIS) lesions, while it was found to be activated in a subset of seminomas and in embryonal carcinomas at the invasive stage [150]. This suggests that, at least in a subset of tumors, R-loops might arise along with persistent replication-associated DNA damage. Their accumulation might contribute to increased genome instability [151] and perhaps tumor progression. Interestingly, by studying a series of nonseminomatous GCTs it has also been found that tumors resistant to cisplatin express low levels of the mammalian serine/arginine-rich protein-specific kinase 1 (SRPK1)[152]. The latter, by phosphorylating the splicing factor SRSF1 promotes its subcellular nuclear localization [153]. Given that inactivation of SRSF1 has been demonstrated to promote genome instability via formation of R-loops [154], one can speculate that low expression of SRPK1 in GCTs may favour genome instability, promoting genome rearrangements leading to the acquisition of resistance to cisplatin.

As the role of R-loops in tumorigenesis will advance in the years to come, and the interactome of DNA:RNA hybrid will expand, it will be interesting to investigate further their potential role in the pathogenesis of TGCTs.

## 4. Conclusions

In summary, numerous observations have showed that mis-regulation of RNA metabolism is implicated in TGCT onset, development and progression, as reported in Table 1. Expression of RNAs species, as sncRNA, influences mRNA stability and translation efficiency. Similarly, lncRNAs regulate gene expression by acting as a molecular sponge of miRNAs and molecular scaffold for protein interaction. Furthermore, increasing evidence has also reported that regulation of transcription-related processes, such as pre-mRNA processing and R-loop resolution, ensures proper gene expression and genome stability. Thus, it is not surprising that dysregulation of these RNAs species contributes to reprogram gene expression favouring TGCTs. Despite the extensive effort made over the course of the last decade to understand the contribution of aberrant regulation of RNA metabolism to TGCT pathogenesis, many molecular details remain poorly understood, thereby leaving many key issues unresolved. For instance, the impact and contribution of many cancer-related non-coding RNAs species to TGCT development is also unknown.

Numerous molecular and biochemical approaches have contributed to the identification of regulatory processes contributing to RNA dysregulation in TGCT. However, these conventional approaches should be flanked by high-throughput technologies allowing the reassessment of single molecule studies in a global cellular environment. Indeed, full sequencing of the whole transcriptome by RNA-sequencing might help to identify differentially expressed genes, novel splice variants and non-coding transcripts at higher resolution. Furthermore, we believe that transcriptome profiling during TGCT progression might help to identify the molecular changes between onset and the late phase of tumor development, providing new targets to develop innovative therapeutic approaches. Indeed, although these tumors show a very high curability, a fraction of treated patients develop drug resistance through molecular mechanisms that are still elusive and that could involve non-coding RNAs. Thus, understanding the functions of RNA molecules in tumors can open new avenues to the identification of novel diagnostic biomarkers or therapeutic targets for TGCTs.

## Figures and Tables

**Table 1:** Altered ncRNAs expression in TGCTs.

RNA Molecule	Expression Level	Function/s	References
**miRNA**			
miR302/367	Upregulated	OncomiR (upregulation of SPRY4 expression and MAPK/ERK pathway)	[29]
miR371/3	Upregulated	OncomiR (downregulation of tumor suppressor LATS2)	[25]
miR223-3p	Upregulated	OncomiR (downregulation of tumor suppressor FBXW7)	[33]
miR449a-b	Downregulated	Tumor suppressor (upregulation of CDK6)	[34]
miR125b	Downregulated	Tumor suppressor (upregulation of tumor-derived chemokine CSF1 and CX3CL1; macrophage recruitment)	[40]
miR506/14	Downregulated	Tumor suppressor (upregulation of PEG and NF-kB pathway)	[35]
Let7/26a	Downregulated	Tumor suppressor (HMGA1 upregulation)	[36]
**piRNA**			
piRNAs	downregulated		[51]
**lncRNA**			
XIST	Upregulated in seminoma		[58]
TDRG1	Upregulated in seminoma	Onco-lncRNA	[59,60,61,62]
H19	upregulated	Onco-lncRNA	[63,64,65,66,67]
SPRY4-IT1	upregulated	Onco-lncRNA	[70]
NLC1-C	accumulated in the nucleus	Onco-lncRNA	[71]
HOTTIP	upregulated	Onco-lncRNA	[73]
**RNA splicing variant**			
MAD2β and MAD2γ	upregulated	Cell cycle progression	[108,109]
Livin α and β	Upregulated in seminoma	Cell survival and apoptosis	[100]

## Data Availability

Not applicable.
